# In Vitro Color Stability Evaluation of Three Polished and Unpolished Nanohybrid Resin Composites Immersed in a 0.12% Chlorhexidine-Based Mouthwash at Different Times

**DOI:** 10.3390/polym15061339

**Published:** 2023-03-07

**Authors:** Reyna Allccahuaman-Avalos, Ramín Medina-Sánchez, Leonor Castro-Ramirez, Marysela Ladera-Castañeda, Luis Cervantes-Ganoza, Reynaldo Martínez-Campos, Fredy Solís-Dante, Ana Aliaga-Mariñas, Arturo Verástegui-Sandoval, César Cayo-Rojas

**Affiliations:** 1 School of Stomatology, Universidad Privada San Juan Bautista, Lima 15067, Peru; 2 Research Team “Salud Pública—Salud Integral”, Faculty of Dentistry, Postgraduate School, Universidad Nacional Federico Villarreal, Lima 15001, Peru; 3 Faculty of Stomatology, Universidad Inca Garcilaso de la Vega, Lima 15084, Peru; 4 Oral Rehabilitation Department, School of Stomatology, Universidad Científica del Sur, Lima 15067, Peru; 5 Faculdade Do Centro Oeste Paulista, Bauru 17012, Brazil; 6 Faculty of Health Sciences, Professional Academic School of Dentistry, Universidad Privada Norbert Wiener, Lima 15046, Peru

**Keywords:** nanohybrid composite, in vitro study, dental materials, dental polishing, resin composite, color variation, chlorhexidine

## Abstract

The use of chlorhexidine-based mouthwashes on resin composites with rough surfaces can cause discoloration which compromises the esthetic of patients. The present study aimed to evaluate the in vitro color stability of Forma (Ultradent Products, Inc., South Jordan), Tetric N-Ceram (Ivoclar Vivadent, Schaan, Liechtenstein) and Filtek Z350XT (3M, ESPE, St. Paul, MN, USA) resin composites, with and without polishing, after being immersed in a 0.12% chlorhexidine (CHX)-based mouthwash at different times. The present in vitro experimental and longitudinal study used 96 nanohybrid resin composite blocks (Forma, Tetric N-Ceram and Filtek Z350XT) 8 mm in diameter and 2 mm thick, evenly distributed. Each resin composite group was divided into two subgroups (n = 16) with and without polishing and then immersed in a 0.12% CHX-based mouthwash for 7, 14, 21 and 28 days. Color measurements were performed with a calibrated digital spectrophotometer. Nonparametric tests were used to compare independent (Mann–Whitney U and Kruskal–Wallis) and related (Friedman) measures. In addition, the Bonferroni post hoc correction was used considering a significance level of *p* < 0.05. All polished and unpolished resin composites presented color variation < 3.3 when immersed for up to 14 days in 0.12% CHX-based mouthwash. The polished resin composite with the lowest color variation (ΔE) values over time was Forma, and the one with the highest values was Tetric N-Ceram. When comparing the color variation (ΔE) over time, it was observed that the three resin composites, with and without polishing, presented a significant change (*p* < 0.001), although these changes in color variation (ΔE) were evident from 14 days between each color acquisition (*p* < 0.05). The unpolished Forma and Filtek Z350XT resin composites showed significantly more color variation than the same polished ones at all times when immersed in a 0.12% CHX-based mouthwash for 30 s daily. In addition, every 14 days, all three resin composites with and without polishing showed a significant color change, while, every 7 days, color stability was maintained. All the resin composites showed clinically acceptable color stability when exposed for up to 14 days to the above-mentioned mouthwash.

## 1. Background

The esthetic expectations of patients have increased over the years, and this has led to an increase in the clinical use of resin composites [1,2,3,4].

The resin composites consist of a resin matrix and inorganic particles that have been chemically and physically designed to meet esthetic requirements. For this reason, they come in different colors or shades that resemble enamel and dentin [2,3,4]. Among its advantages as a restorative material is its chromatic similarity to teeth. [5].

One of the most crucial success criteria for restorative dentistry is to ensure long-term color stability and harmony [5,6]. In recent years, there have been improvements in resin composite formulation mainly through the use of nanotechnology. Nanohybrid resin composites contain nanoscale inorganic particles dispersed in the resin matrix that result in a more polished surface, less shrinkage, color stability and improved esthetics [3,4,7]. However, despite the advancement in resin composite technology, a major problem is color stability in the oral cavity [1,5,8].

Color variations can be caused by intrinsic or extrinsic factors. Intrinsic factors originate from the material’s own structure, such as resin matrix, filler weight, particle size or photoinitiator type. Extrinsic factors include the absorption of dyes from external sources such as nicotine, coffee, tea, wine and mouthwashes such as chlorhexidine [2,3,5,6]. The latter extrinsic discoloration is the most significant factor affecting color stability and long-term durability [5].

Chlorhexidine is a biguanide compound prescribed by dentists due to its bactericidal properties and effective anti-plaque action which blocks the free acid groups (sulfates, carboxyls and phosphates), favoring the non-adhesion and co-aggregation of bacteria. Chlorhexidine also binds to the negative charges on the bacterial cell wall, hindering the adhesion mechanism between them. However, this composite is also relevant in terms of color stability due to its chromogenic potential, causing brown stains on the teeth, tongue and on silicate and resin composite restorations [9]. Several staining mechanisms have been described for chlorhexidine such as its degradation to release parachloraniline, non-enzymatic browning reactions, denaturation of proteins by chlorhexidine with formation of metal sulfide and precipitation of anionic dietary chromogens by cationic antiseptics [3,6,10].

The prescription of chlorhexidine-based mouthwashes has become common for the control of periodontal disease, periodontal surgeries and to improve wound healing after surgical procedures in the oral environment. Furthermore, in the context of the COVID-19 pandemic, CHX is still frequently prescribed together with cetylpyridinium chloride [1,3]. CHX present in mouthwashes could affect the color stability of resin composite restorations [9], even more so if other factors contribute to pigment retention on the resin composite surface. Therefore, a coarse-to-fine-grained polishing system needs to be applied in order to test whether a considerable color variation in nanohybrid resin composites can be avoided [5,10,11].

Therefore, the present study aimed to evaluate the in vitro color stability of Forma, Tetric N-Ceram and Filtek Z350XT resin composites, with and without polishing, after being immersed in 0.12% chlorhexidine-based mouthwash at different times. The null hypothesis was that Forma, Tetric N-Ceram and Filtek Z350XT resin composites, with and without polishing, would not show significant differences in their in vitro color stability after immersion in 0.12% chlorhexidine-based mouthwash at different times.

## 2. Materials and Methods

### 2.1. Type of Study and Delimitation

This experimental in vitro and longitudinal study was performed in the Dent Import laboratory, Lima, Peru, from February to March 2022. This study was exempted from protocol review by an institutional ethics committee; however, it issued a letter of authorization for the execution of project no. 114-2022-CIEI-UPSJB. In addition, this study considered the CRIS guideline (‘Checklist for Reporting In-vitro Studies’) [12].

### 2.2. Sample Calculation and Selection

**Figure 1:**
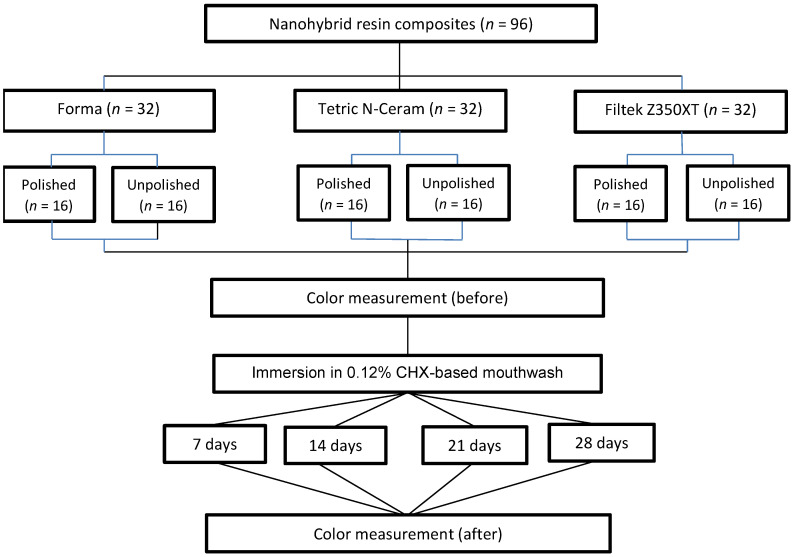
Random distribution of groups according to type of resin composite, 0.12% chlorhexidine immersion and with/without polishing.

Ninety-six blocks of resin composites were made and standardized. They were evenly distributed into three groups of 32 resin blocks and then subdivided in a simple, random fashion without replacement into two equal groups of polished (n = 16) and unpolished (n = 16) resin blocks (Figure 1). The total sample size (n = 96) was calculated based on data obtained in a previous pilot study where the formula for analysis of variance was applied using G*Power statistical software version 3.1.9.7 considering a significance level of (α) = 0.05, a statistical power of (1 − β) = 0.80 and an effect size of 0.28 with 6 groups and 4 paired measures.

### 2.3. Sample Characteristics and Preparation

A silicone mold measuring 8 mm in diameter × 2 mm thick was used to make the resin composite blocks (Table 1) [1,5]. An incremental technique was used by placing 2 mm layers of resin on a glass base using a TNPFIW3 spatula (Hu-Friedy, Chicago, IL, USA). Each layer was light-cured with an LED (light-emitting diode) lamp (Valo^®^, Ultradent, South Jordan, UT, USA) at a power of 1000 mW/cm^2^ for 20 s [13]. The intensity was checked with a radiometer (Litex 682, Dentamerica^®^, City of Industry, CA, USA). The last resin layer was varnished with glycerin before the final light-curing in order to avoid the inhibited oxygen layer [14,15]. Then, each group of resin composites (n = 16) was polished by the same operator for 20 s per step according to the manufacturer’s indication. A four-step coarse-to-fine-grit disc system (Sof-Lex, 3M ESPE, St. Paul, SM, USA) [16] with an electric motor (EM-E6, W&H, Bürmoos, Austria) and a contra-angle handpiece (NSK, Tokyo, Japan) was used at a speed of 15,000 rpm with identical movements and in the same direction. The samples were then washed and dried to remove surface residues.

### 2.4. Color Variation Measurement

The color of the 96 resin composite blocks was measured with a calibrated spectrophotometer (Vita Easyshade^®^, V Zahnfabrik, Bad Säckingen, Germany) according to ISO/TR 28642:2016 and the CIELAB scale [17], obtaining a measurement for the individual color coordinates (L*, a* and b*) representing the luminance value, red/green value and blue/green value, respectively. The measurement was performed twice for each sample, and the device was calibrated according to the manufacturer’s instructions after each measurement. The probe tip was placed perpendicular and tightly fitted to the samples’ surface for accurate measurements. A black box was used for sample positioning with standardized site, angle and surrounding illumination during measurements. After this process, the samples were stored in closed and labeled glass jars with distilled water for 24 h post polymerization. Then, they were immersed in 20 mL of a mouthwash based on chlorhexidine 0.12% + cetylpyridinium chloride 0.05% (Perio-Aid^®^, Dentaid, Lima, Peru) for 30 s per day, as recommended by the Food and Drug Administration (FDA) [1,3]. Subsequently, color stability was measured with the same calibrated spectrophotometer at 7 days, 14 days, 21 days and 28 days after the samples were washed with distilled water and dried with absorbent paper. All measurements were performed in the same environment and by the same operator. The CIEDE2000 color system and the following formula were used to evaluate the color variation:

(1) $$\Delta E_{00}={\left[{\left(\frac{\Delta L}{K_{L}S_{L}}\right)}^{2}+{\left(\frac{\Delta C}{K_{C}S_{C}}\right)}^{2}+{\left(\frac{\Delta H}{K_{H}S_{H}}\right)}^{2}+R_{T}\left(\frac{\Delta C}{K_{C}S_{C}}\right)\left(\frac{\Delta H}{K_{H}S_{H}}\right)\right]}^{1/2}$$

where ΔL, ΔC and ΔH represent the differences in luminance, chroma and hue, respectively, between the initial and subsequent color measurements. SL, SC and SH are the weight functions incorporated into the formula to eliminate irregularities observed in the CIE system. L*, a*, b* refer to brightness, color density and hue, respectively. For RT, a value of 0 (ΔC = 0) is assumed for colors falling within the same color density radius. KL, KC and KH are parametric factors calculated for brightness, color chromaticity and hue, respectively, and were included in the formula to correct for errors arising from experimental conditions such as the surface of a material and the background against which a measurement was made [6], all in accordance with ISO/CIE11664-6:2020 [18].

### 2.5. Statistical Analysis

SPSS software (Statistical Package for the Social Sciences, IBM, NY, USA) version 28.0 was used for data analysis. For descriptive analysis, the mean, median, standard deviation and interquartile range were calculated. For hypothesis testing, the normality and homogeneity of variances were verified with the Shapiro–Wilk test and Levene’s test, respectively. According to the results, normality of the data was not observed, so it was decided that the nonparametric Mann–Whitney U test was to be used to compare two independent measures and the Kruskal–Wallis test to compare more than two independent measures. The Friedman test was used to compare more than two related measures according to time. In addition, the Bonferroni post hoc correction was used if significant differences were detected in both the Kruskal–Wallis test and the Friedman test. A significance level of *p* < 0.05 was considered in all comparisons.

## 3. Results

When comparing the color variation (ΔE) of the polished and unpolished resin composites, it was observed that the Tetric N-Ceram resin composite only presented significant differences at 21 days after being immersed in 0.12% chlorhexidine (*p* = 0.019). The unpolished Forma and Filtek Z350XT resin composites presented significantly greater color variation (ΔE) with respect to the same polished ones (*p* < 0.05) at all the times analyzed (7, 14, 21 and 28 days) (Table 2).

With respect to the polished resin composites immersed in 0.12% chlorhexidine, no significant differences in color variation (ΔE) (*p* = 0.701) were observed at 7 days. However, at 14 days, it was observed that the Forma resin composite showed significantly lower color variation (ΔE) compared to the Tetric N-Ceram and Filtek Z350XT resin composites (*p* = 0.012 and *p* = 0.011, respectively). These last two resin composites did not differ significantly from each other (*p* > 0.05). Finally, at 21 and 28 days, the Tetric N-Ceram resin presented significantly greater color variation (ΔE) with respect to the Forma (*p* < 0.001 and *p* < 0.001, respectively) and Filtek Z350XT (*p* < 0.012 and *p* < 0.007, respectively) resin composites; however, these two resin composites did not differ significantly from each other at either time (*p* > 0.05) (Table 3 and Table 4).

Regarding the unpolished resin composites immersed in 0.12% chlorhexidine for 7 days, a significantly greater color variation (ΔE) was observed for the Forma resin composite compared to for the Tetric N-Ceram and Filtek Z350XT resins (*p* < 0.001 and *p* = 0.001, respectively); however, the latter two did not differ significantly from each other (*p* > 0.05). In addition, the Filtek Z350XT resin composite showed significantly higher color variation (ΔE) than the Tetric N-Ceram resin composite at 14 and 21 days (*p* = 0.012 and *p* = 0.028, respectively). The Forma resin composite at 14 and 21 days did not show significant differences compared with the other resin composites (*p* > 0.05). Finally, at 28 days, the Filtek Z350XT resin composite showed significantly greater color variation (ΔE) with respect to the Forma resin composite (*p* = 0.035) but not with respect to the Tetric N-Ceram resin composite (*p* = 0.326) (Table 3 and Table 4).

**Figure 2:**
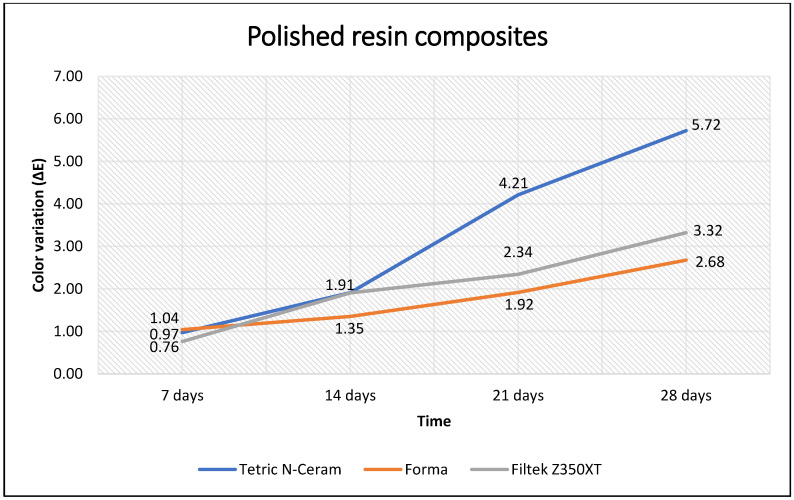
Color variation (ΔE) of polished resin composites according to immersion time.

**Figure 3:**
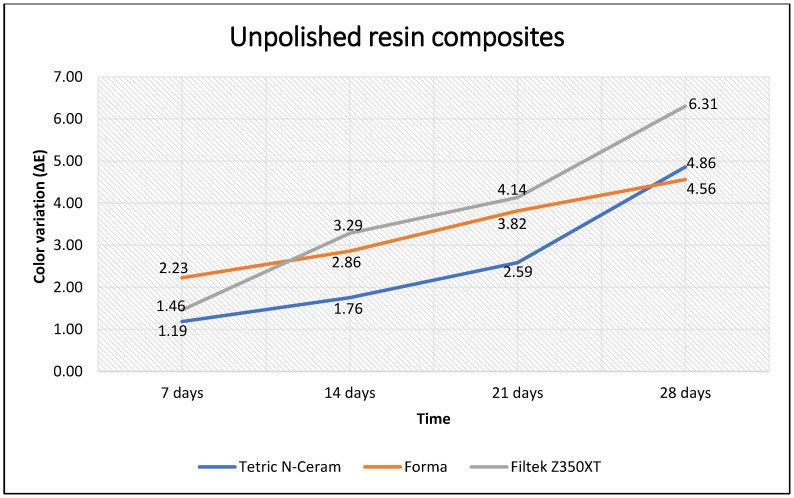
Color variation (ΔE) of unpolished resin composites according to immersion time.

When comparing the color variation (ΔE) over time, it was observed that the three resin composites with and without polishing showed significant variation (*p* < 0.001). These changes in color variation (ΔE) remained significant after 14 days between each color measurement (*p* < 0.05). When measured at 7 days, these changes in color variation (ΔE) were not significant (*p* > 0.05) (Table 5). Furthermore, it was observed that the polished resin composite with the lowest values of color variation (ΔE) over time was Forma, and the polished resin composite with the highest values was Tetric N-Ceram. Contrarily, when the Tetric N-Ceram resin composite was polished, it maintained the lowest color variation (ΔE) values most of the time, while the unpolished Filtek Z350XT resin composite maintained the highest values (Figure 2 and Figure 3).

## 4. Discussion

Color change or variation is one of the main reasons for the replacement of restorations, especially in anterior teeth [9,17,19]. The discoloration of resin composites has a multifactorial etiology, with chlorhexidine-based mouthwashes being one of the causative factors [2,6,9]. The present study aimed to evaluate the in vitro color stability of Forma, Tetric N-Ceram and Filtek Z350XT nanohybrid resin composites, with and without polishing, after being immersed in a 0.12% chlorhexidine-based mouthwash at different times. As a result, the null hypothesis was rejected.

The results in the present study showed that the polished Tetric N-Ceram resin composite presented greater color variation compared to the Filtek Z350XT and Forma resin composites after being immersed for 21 and 28 days in 0.12% chlorhexidine-based mouthwash. In addition, the unpolished Filtek Z350XT and Forma resin composites showed greater color variation compared to the same polished resins at all times analyzed. The three resin composites with and without polishing showed a significant color variation within 14 days, while color stability was maintained within 7 days. All these obtained results are in agreement with the results of Hasani et al. [1], Kroskavi et al. [3], Zajkani [5], Shabika et al. [20] and Salman et al. [21]. This possibly suggests that the use of mouthwashes for more than one week may alter the shade stability of conventional nanohybrid resin composites. The literature establishes a clinically acceptable value of ΔE ≤ 3.3 [5,8,19,22]. Therefore, in the present study, the color variations of the submerged resin composites between 7 and 14 days, with and without polishing, were considered clinically acceptable, while, at 21 and 28 days, they presented significant color variation in most groups. These findings may indicate that the use of 0.12% chlorhexidine-based mouthwash for three or more weeks may not only alter the color stability of the resin composites, but the variations may even be clinically unacceptable. The dentist should weigh the risk/benefit before prescribing this mouthwash for a prolonged period of time in patients with resin composite restorations and avoid the need to renew restorations due to discoloration [6].

At most of the times evaluated, the unpolished Tetric N-Ceram resin composite showed significant differences in color change with respect to the same polished one. The Forma and Filtek Z350XT polished resin composites showed significant color changes in all the times evaluated with respect to the same ones without polishing. This may be because the roughness of their surface due to lack of polishing makes them susceptible to pigment retention and color variation [17,23]. At 28 days, all polished and unpolished resin composites showed color variation above the clinical standard with the exception of the polished Forma and Filtek Z350XT resin composites.

Currently, the use of nanofilled resin composites is increasing due to their significant improvement in filler size, low wear and high resistance against degradation [3,5]. In addition, smaller filler size can contribute to stain reduction and improve esthetic appearance [4,5,7]. This is important to keep in mind as it has been reported that the size and distribution of filler can be related to color changes [3,5]. In addition, some reports have related external discoloration of the resin composite to large filler particles as they provide increased surface roughness [17,24].

The color change in the resin composite samples used may also be associated with the different compositions as they differ in their chemical formulations [5,6,25,26]. The presence of triethylene glycol-dimethacrylate (TEGDMA) within the resin composite composition can lead to increased water absorption and staining rates, causing the adsorbed water to induce hydrolytic degradation or detachment of the filler matrix [1,27,28]. Zhang et al. [23] reported that water absorption of polymers can cause softening of the resin composite matrix components, thus, reducing the resistance to discoloration. In contrast, urethane dimethacrylate (UDMA) represents less risk to color changes due to its lower viscosity, low water absorption and better polymerization compared to other methacrylate-based monomers [1,5,6]. The TEGDMA in the composition of Forma and Filtek Z350XT could explain the clinically unacceptable color changes presented by these resin composites at both 21 and 28 days when they were not polished. However, with these same chlorhexidine immersion times, both polished resin composites showed clinically acceptable shade stability (ΔE ≤ 3.3). Considering the small size of Filtek Z350XT (5–20 nm) and Forma (5–50 nm) filler particles, it was confirmed that the size and distribution of the fillers may be related to color variation as well as better polishability, leading to better color stability [3,5]. This would also explain why the Tetric N-Ceram resin composite was more susceptible to color variation since it contained larger filler particles (40–3000 nm) with higher water absorption and higher surface roughness, resulting in higher discoloration despite polishing.

In recent years, the prescription of mouthwashes has become common. Chlorhexidine as an antiseptic solution has been used for the control of periodontal disease, in periodontal surgeries and also to improve healing after surgical procedures in the oral environment. Furthermore, in the context of the COVID-19 pandemic, CHX is frequently prescribed together with cetylpyridinium, as it has been reported to be able to decrease the SARS-CoV-2 viral load [1,3,29]. The presence of cetylpyridinium did not influence the study because it has been shown to have fewer side effects compared to chlorhexidine, as, in the study by Rahman et al. [30], they reported color changes only in subjects using chlorhexidine-based rinses, while no color variation was reported with cetylpyridinium. Therefore, in the present study it was decided to evaluate the pigmenting effect of Perio-Aid^®^ on three nanohybrid resin composites over time [1,3]. However, adverse effects of CHX present in mouthwashes have been reported (dysgeusia and dental pigmentation being among the most common) depending on its dosage. This has led to the suggestion over the years of different CHX concentrations to balance beneficial and adverse effects in order to improve patient treatment [24]. The Food and Drug Administration (FDA) suggests the use of CHX as a mouthwash in 10 to 20 mL presentations with concentrations of 0.12% and 0.2% for approximately 30 s and for a period that can vary between 2 and 4 weeks [1,3]. For this reason, it was decided to evaluate the color stability of resin composites exposed to a chlorhexidine-based mouthwash for up to 28 days.

The use of a spectrophotometer to objectively assess color variation is a strength of the present study design, as it minimizes information bias compared to assessing color variation by visual orientation [15,21,22,25]. It is also important to emphasize that the present study evaluated the nanohybrid resin composite brands most commonly used in dental esthetics. This will help the dentist to make a decision when considering the prescription of 0.12% chlorhexidine-based mouthwashes. Furthermore, the choice of the Sof-Lex polishing disc system was based on its reported considerable decrease in resin surface roughness compared to other polishing systems [29,30,31,32,33]. This system was also used as the standard protocol because of its ability to form smooth surfaces that are less susceptible to chemical solubility [34].

As a recommendation, dentists should promptly and clearly inform their patients about the clinically unacceptable color change that may occur in resin composite restorations when they are exposed to 0.12% chlorhexidine mouthwash treatments for a period longer than 14 days. According to the results obtained, this period would be the recommended one to maintain the chromatic stability of resin composites within the clinical standard. It is advisable to add the use of cetylpyridinium chloride (CPC) as a control group to the study design in the case of evaluating the pigmenting effect of Perio-Aid^®^ because CPC can also cause staining but to a lesser degree than chlorhexidine [35,36]. As a limitation, it should be recognized that the results of the present in vitro study cannot be fully extrapolated to the clinical field due to the different factors that may affect the color stability of restorative materials in the oral cavity such as the presence of saliva, biofilm and the effect of different foods and beverages that are difficult to simulate in an in vitro environment [2,3]. Another limitation to mention is the immersion method for the samples as it could not accurately reflect the effects of the intermittent use of mouth rinses. The design of randomized and controlled clinical studies is recommended in order to determine the degree of discoloration caused by chlorhexidine-based mouthwashes in the oral environment [6]. Finally, taking into account the above limitations and the parameters used in the present study, further research is recommended to evaluate the color variation in bulk-fill resin composites under various conditions considering other polishing methods, mouth rinses with different composition and different immersion times.

## 5. Conclusions

The unpolished Forma and Filtek Z350XT resin composites showed significantly greater color variation than the same polished composite resins at 7, 14, 21 and 28 days of exposure to 0.12% CHX-based mouthwash for 30 s per day. At 7 days, these two resins and the Tetric N-Ceram, all polished, showed no difference in color variation. Furthermore, every 14 days, the three resin composites with and without polishing showed a significant color change, while, every 7 days, color stability was maintained. All resin composites showed clinically acceptable color stability after being exposed for up to 14 days to the above-mentioned mouthwash.

## Figures and Tables

**Table 1:** Technical profile of products used.

Product	Type	Composition	Filler % (wt% vol%)	Manufacturer	Lot
Filtek™ Z350XT A1	Nanohybrid	Bis-GMA, Bis-EMA, UDMA, PEGDMA TEGDMA (CQ) Zirconia/silica cluster and silica nanoparticle	78.5 wt% 63.3 vol%	3M, ESPE, St. Paul, MN, USA	69,560
Tetric^®^ N-Ceram A1	Nanohybrid	Bis-GMA Bis-EMA UDMA (CQ) Barium glass, ytterbium trifluoride, mixed oxide, silicon dioxide, prepolymers	81.2 wt% 57 vol%	Ivoclar Vivadent, Schaan, Liechtenstein	Z029G9
Forma™ A1B	Nanohybrid	Bis-GMA, TEGDMA, Bis-EMA and UDMA Ytterbium trifluoride, zirconia/silica inorganic filler combination and barium glass	67 wt% vol% not disclosed by manufacturer	Ultradent Products, Inc., South Jordan, UT, USA	D0GJX
Sof-Lex System	Finishing Polishing System	Aluminum oxide abrasive discs	SL coarse: 60 μm SL medium: 29 μm SL fine: 14 μm SL superfine: 5 μm	3M, ESPE, St. Paul, MN, USA	N980358 N952113 N960093 N951874

**Table 2:** Color variation (ΔE) comparison of each resin composite with and without polishing according to time.

Time	Polish	n	Tetric N-Ceram					Forma					Filtek Z350XT				
			Mean	SD	Median	IQR	* *p*	Mean	SD	Median	IQR	* *p*	Mean	SD	Median	IQR	* *p*
**7 days**	**Yes**	**16**	**1.10**	**0.72**	**0.97**	**1.06**	**0.590**	**0.99**	**0.34**	**1.04**	**0.55**	**<0.001**	**0.91**	**0.49**	**0.76**	**0.74**	0.047
	No	16	1.12	0.39	1.19	0.67		2.29	0.46	2.23	0.66		1.39	0.68	1.46	1.07	
**14 days**	Yes	16	2.17	1.06	1.91	1.45	0.696	1.32	0.43	1.35	0.60	<0.001	1.89	0.45	1.91	0.71	<0.001
	No	16	2.10	1.19	1.76	1.72		2.92	0.41	2.86	0.60		3.13	0.96	3.29	1.93	
**21 days**	Yes	16	4.10	1.47	4.21	2.57	0.019	1.92	0.64	1.92	1.06	<0.001	2.31	0.42	2.34	0.63	<0.001
	No	16	2.85	1.50	2.59	2.49		3.83	0.72	3.82	0.93		4.06	0.95	4.14	1.40	
**28 days**	Yes	16	5.69	1.22	5.72	1.83	0.056	2.81	0.73	2.68	1.07	<0.001	3.83	1.31	3.32	2.33	0.002
	No	16	4.90	0.58	4.86	0.90		4.46	1.09	4.56	1.56		6.21	2.15	6.31	3.42	

n: sample size; SD: standard deviation; IQR: interquartile range; * based on Mann–Whitney U test, *p* < 0.05 (significant differences).

**Table 3:** Color variation (ΔE) comparison between polished and unpolished resin composites according to time.

Time	Resin Composite	n	Polished					Unpolished				
			Mean	SD	Median	IQR	* *p*	Mean	SD	Median	IQR	* *p*
**7 days**	Tetric N-Ceram	16	1.10	0.72	0.97 ^A^	1.06	0.701	1.12	0.39	1.19 ^A^	0.67	<0.001
	Forma	16	0.99	0.34	1.04 ^A^	0.55		2.29	0.46	2.23 ^B^	0.66	
	Filtek Z350XT	16	0.91	0.49	0.76 ^A^	0.74		1.39	0.68	1.46 ^A^	1.07	
**14 days**	Tetric N-Ceram	16	2.17	1.06	1.91 ^A^	1.45	0.004	2.10	1.19	1.76 ^A^	1.72	0.011
	Forma	16	1.32	0.43	1.35 ^B^	0.60		2.92	0.41	2.86 ^A,B^	0.60	
	Filtek Z350XT	16	1.89	0.45	1.91 ^A^	0.71		3.13	0.96	3.29 ^B^	1.93	
**21 days**	Tetric N-Ceram	16	4.10	1.47	4.21 ^A^	2.57	<0.001	2.85	1.50	2.59 ^A^	2.49	0.022
	Forma	16	1.92	0.64	1.92 ^B^	1.06		3.83	0.72	3.82 ^A,B^	0.93	
	Filtek Z350XT	16	2.31	0.42	2.34 ^B^	0.63		4.06	0.95	4.14 ^B^	1.40	
**28 days**	Tetric N-Ceram	16	5.69	1.22	5.72 ^A^	1.83	<0.001	4.90	0.58	4.86 ^A,B^	0.90	0.038
	Forma	16	2.81	0.73	2.68 ^B^	1.07		4.46	1.09	4.56 ^A^	1.56	
	Filtek Z350XT	16	3.83	1.31	3.32 ^B^	2.33		6.21	2.15	6.31 ^B^	3.42	

n: sample size; SD: standard deviation; IQR: interquartile range; * based on Kruskal–Wallis H test, *p* < 0.05 (significant differences); ^A^ and ^B^: different letters in each column of the median according to time indicate significant differences (*p* < 0.05) based on Dunnett’s post hoc test with Bonferroni correction.

**Table 4:** Multiple comparison of color variations (ΔE) between polished and unpolished resin composites as a function of time.

Time	Resin Composite	Polished		Unpolished	
		Forma	Filtek Z350XT	Forma	Filtek Z350XT
**7 days**	Tetric N-Ceram			*p* < 0.001 *	*p* = 0.721
	Forma				*p* = 0.001 *
**14 days**	Tetric N-Ceram	*p* = 0.012 *	*p* = 1.000	*p* = 0.077	*p* = 0.012 *
	Forma		*p* = 0.011 *		*p* = 1.000
**21 days**	Tetric N-Ceram	*p* < 0.001 *	*p* = 0.012 *	*p* = 0.102	*p* = 0.028 *
	Forma		*p* = 0.399		*p* = 1.000
**28 days**	Tetric N-Ceram	*p* < 0.001 *	*p* = 0.007 *	*p* = 1.000	*p* = 0.326
	Forma		*p* = 0.180		*p* = 0.035 *

* Based on Dunnett’s post hoc test with Bonferroni correction (*p* < 0.05, significant differences).

**Table 5:** Color variation (ΔE) comparison over time according to the type of resin composite with and without polishing.

Resin Composite	Polish	n	7 days		14 days		21 days		28 days		* *p*
			Median	IQR	Median	IQR	Median	IQR	Median	IQR	
**Tetric N-Ceram**	Yes	16	0.97 ^A^	1.06	1.91 ^A,B^	1.45	4.21 ^B,C^	2.57	5.72 ^C^	1.83	<0.001
	No	16	1.19 ^A^	0.67	1.76 ^A,B^	1.72	2.59 ^B,C^	2.49	4.86 ^C^	0.90	<0.001
**Forma**	Yes	16	1.04 ^A^	0.55	1.35 ^A,B^	0.60	1.92 ^B,C^	1.06	2.68 ^C^	1.07	<0.001
	No	16	2.23 ^A^	0.66	2.86 ^A,B^	0.60	3.82 ^B,C^	0.93	4.56 ^C^	1.56	<0.001
**Filtek Z350XT**	Yes	16	0.76 ^A^	0.74	1.91 ^A,B^	0.71	2.34 ^B,C^	0.63	3.32 ^C^	2.33	<0.001
	No	16	1.46 ^A^	1.07	3.29 ^A,B^	1.93	4.14 ^B,C^	1.40	6.31 ^C^	3.42	<0.001

n: sample size; IQR: interquartile range; * based on Friedman’s test, *p* < 0.05 (significant differences); ^A^, ^B^ and ^C^: different letters in the median of each row of the resin composites indicate significant differences (*p* < 0.05) based on Bonferroni post hoc correction.

## Data Availability

The data presented in this study are available on request from the corresponding author.
